# Editorial: Optogenetics: An Emerging Approach in Cardiac Electrophysiology

**DOI:** 10.3389/fphys.2020.00414

**Published:** 2020-04-28

**Authors:** Christopher L.-H. Huang, Emily A. Ferenczi, Ming Lei

**Affiliations:** ^1^Physiological Laboratory and Department of Biochemistry, University of Cambridge, Cambridge, United Kingdom; ^2^Key Laboratory of Medical Electrophysiology of the Ministry of Education and Institute of Cardiovascular Research, Southwest Medical University, Luzhou, China; ^3^Department of Neurology, Harvard Medical School, Massachusetts General Hospital and Brigham and Women's Hospital, Boston, MA, United States; ^4^Department of Pharmacology, University of Oxford, Oxford, United Kingdom

**Keywords:** optogenetics, cardiac electrophysiology, cardiac arrhythmias, clinical translation, opsins

Optogenetic techniques modulate excitable membranes through activation of photosensitive opsin proteins functioning as light-gated channels, transporters or receptors. Of the known, prokaryotic, algal or fungal, type I, and animal, type II, opsins, the type I opsins have been the most frequently used experimentally since they were first to be expressed in mammalian neurons in the early 2000s. Optogenetic approaches circumvent disadvantages of traditional, direct electrical stimulation, methods, such as tissue toxicity, and enhance cellular and spatial specificity of stimulus effects through genetic or developmental targeting.

These methods initially found neuroscience applications and these ranged widely from use in *in vitro* models to behaving animals, and in invertebrate through to mammalian systems (Boyden et al., 2005). They have more recently proved relevant to other excitable, particularly cardiac, tissues, in the latter case, particularly in fundamental scientific and translational studies of cardiac arrhythmogenesis (Bruegmann et al., 2016). Arrhythmias are the result of disruption of the normally orderly electrical excitation sequence initiating co-ordinated and effective atrial or ventricular contraction. They account for ~3.7 million human deaths/year worldwide (Kuriachan et al., 2017). Their clinical management has often lagged progress in many other cardiological areas. This likely reflects our currently limited understanding of the physiological mechanisms that underly their initiation, maintenance or propagation (Huang, 2017; Lei et al., 2018; Huang et al., 2020).

Cardiac optogenetic approaches offer unprecedented opportunities and new exciting possibilities to resolving and intervening in such normal and diseased function. Optogenetic sensors can be incorporated into specific motifs and targeted at specific, tissue and cell types, and subcellular domains, and applied at high spatial and temporal resolution. This Research Topic collects articles focusing on optogenetic applications in cardiac electrophysiology. They particularly consider their ability to modify membrane voltage, intracellular Ca^2+^ and cellular signaling, and their potential for use in cardiac pacing, cardioversion, cell communication, in arrhythmia research (Boyle et al., 2018). Pioneering contributors in this cardiac field summarize underlying concepts, existing optogenetic tools and approaches, discussing their suitability for cardiac experimental studies and interventions. They then review examples of applications and situations where optogenetics have yielded new insights into and potential interventional approaches for cardiac physiology and cardiac disease.

Two background articles explore *available optogenetic approaches*, opsin proteins and their different ion conductances, kinetics, light sensitivity, and activation spectra, both in general, and bearing on cardiac applications (Ferenczi et al.; O'Shea et al.). These include the physical and practical background, and choices of opsin tools appropriate to the cellular physiology and experimental system in question (Deisseroth, 2015). Cardiac expression of the chosen optogenetic tool is variously achieved by coupling of “spark cells” at the single-cell level, construction of transgenic mice or *in vivo* introduction of adenoviral expression systems at the systems level. Adeno-associated viruses (AAVs) offer advantages in long-term, cardiac-specific gene expression. An article exploring optimal AAV serotypes (1, 6, or 9) reports preferential efficiency of AAV1/6 *in vitro* and AAV9 *in vivo* for cardiac optogenetic delivery (Ambrosi et al.). Optogenetic stimulation either activates or silences cellular excitation through membrane depolarization or hyperpolarization. Depolarizing, activated, cation-conducting channelrhodopsins (ChR) trigger cardiomyocyte action potentials; hyperpolarization by Cl^−^ pumps suppresses activity (Arrenberg et al., 2010). The activated photosensitive Cl^−^ channel, Guillardia theta ChR (GtACR1) silences neuronal action potential generation: microelectrode and *in vivo* optical whole heart recordings demonstrate depolarized cells as expected from the cardiac Cl^−^ Nernst potential, inhibiting re-excitation (Kopton et al.).

Optogenetic techniques promise to deliver *unique physiological insights* unobtainable from established procedures. At the cellular level, it could demonstrate, localize, and characterize electrical and Ca^2+^ transient activity in particular phenylethanolamine-N-methyl transferase (Pnmt)-derived cardiomyocyte subpopulations and their roles in localized adrenergic signaling (Fan et al.). At the whole heart level, optogenetic methods permitted temporally precise, acute, neuron-specific, parasympathetic stimulation in mice expressing ChR2 in their peripheral cholinergic neurons. This elicited sudden, dramatic but reversible heart rate reductions and atrioventricular conduction delays (Moreno et al.).

Optogenetic techniques can target and stably deliver perturbing light stimuli to *specific cell types* at the cellular, organ or whole organism levels, in different cardiac, sino-atrial nodal, atrial, ventricular regions, or the conducting tissues within or between them, permitting focussed study of the entire range of clinically important arrhythmic conditions. Thus, protocols combining optogenetic stimulation with dual cytosolic Ca^2+^- membrane voltage optical mapping studied membrane potential, intracellular Ca^2+^ transients and pacemaker activity in a murine sino-atrial preparation (Dong et al.). Use of pseudo two-dimensional murine ventricular tissue slices profiled transmural and regional voltage and Ca^2+^ signaling gradients under stress produced by high frequency pacing, β-adrenergic stimulation or pathological, including hypertrophic, conditions (He et al.). We also include one report where the genetically-encoded sensor for altered cellular glutathione redox status, Grx1-roGFP2, provided readouts of cellular reactive oxygen species (Trautsch et al.). The latter modulate ryanodine receptor-mediated sarcoplasmic reticular Ca^2+^ release (Prosser et al., 2011) and phospholamban phosphorylation-regulated diastolic relaxation (Scotcher et al., 2016).

We complete the Research Topic by considering translational exploitations of optogenetic methods in *clinical cardiology*. These could achieve non-damaging optical control of excitation with high temporal and spatial resolution for sino-atrial pacemaking, or atrial or ventricular cardioversion. In contrast, normally used electrical shocks do not fully control the spatial extent and level of their resulting membrane potential changes. One article summarizes recent progress on optogenetic defibrillation and cardioversion, their underlying mechanisms and potential patient populations that might thus benefit (Sasse et al.). In channelrhodopsin-2 (ChR2) transgenic mice, a single 10–1,000 ms pulse at light intensities <1.10 mW mm^−2^ sufficed to cardiovert fibrillating hearts into sinus rhythm. Optical mapping attributed this to both annihilating, excitation wave, collisions and propagation perturbations (Quiñonez Uribe et al.). Epicardial illumination terminated ventricular arrhythmias in explanted, hypokalaemic hearts expressing the light-driven proton pump ArchT, perfused with the K_ATP_ opener pinacidil. This was accompanied by hyperpolarization, more rapid action potential upstrokes but reduced conduction velocity implicating an enhanced electrical sink in terminating the arrhythmia. Such novel insights could improve our mechanistic understanding and treatment strategies for arrhythmia termination (Funken et al.).

## Author Contributions

EF surveyed and summarized all the contributions in this Research Topic and ML revised these, permitting CH to formulate the narrative underlying this Research Topic.

## Conflict of Interest

The authors declare that the research was conducted in the absence of any commercial or financial relationships that could be construed as a potential conflict of interest.
